# Being Heard, Exerting Influence, or Knowing How to Play the Game? Expectations of Client Involvement among Social and Health Care Professionals and Clients

**DOI:** 10.3390/ijerph17165653

**Published:** 2020-08-05

**Authors:** Elina Weiste, Sari Käpykangas, Lise-Lotte Uusitalo, Melisa Stevanovic

**Affiliations:** 1 Finnish Institute of Occupational Health, P.O. Box 40, FI-00032 Helsinki, Finland; sari.kapykangas@ttl.fi; 2 Faculty of Social Sciences, University of Helsinki, P.O. Box 54, FI-00014 Helsinki, Finland; lise-lotte.uusitalo@helsinki.fi (L.-L.U.); melisa.stevanovic@helsinki.fi (M.S.)

**Keywords:** client involvement, client participation, cultural change, co-development, conversation analysis, social and health care professionals, interaction, qualitative research

## Abstract

Contemporary social and health care services exhibit a significant movement toward increasing client involvement in their own care and in the development of services. This major cultural change represents a marked shift in the client’s role from a passive patient to an active empowered agent. We draw on interaction-oriented focus group research and conversation analysis to study workshop conversations in which social and health care clients and professionals discussed “client involvement”. Our analysis focuses on the participants’ mutually congruent or discrepant views on the topic. The professionals and clients both saw client involvement as an ideal that should be promoted. Although both participant groups considered the clients’ experience of being heard a prerequisite of client involvement, the clients deviated from the professionals in that they also highlighted the need for actual decision-making power. However, when the professionals invoked the clients’ responsibility for their own treatment, the clients were not eager to agree with their view. In addition, in analyzing problems of client involvement during the clients’ and professionals’ meta-talk about client involvement, the paper also shows how the “client involvement” rhetoric itself may, paradoxically, sometimes serve to hinder here-and-now client involvement.

## 1. Introduction

Cultural change is an opportunity. A culture refers to “a shared set of ideas, norms, and behaviours common to a group of people inhabiting a geographic location” [1], and cultural change makes it possible for people to remove those social and cultural deficits that have led to a repression of certain parts of the population [2]. Cultural change is nonetheless always a complex, multifaceted phenomenon. It is inherently threatening and psychologically stressful in that it introduces more variation to the basic assumptions that underlie people’s actions [3]. Cultural change is associated with various contradictions, such as those between values and practices [4,5], which in turn may be caused by society changing more rapidly than specific organizations and institutions [6]. Allowing and creating space for negotiation has thus been promoted as a significant way to deal with the challenging situations of cultural change [5].

One major cultural change that has recently taken place in the context of social and health care services is related to the involvement of clients in their own care, as well as in the planning, development, and evaluation of services. The notion of client involvement entails the client’s right to be informed about issues relating to them, the opportunity to express their opinion when decisions about their own care are made, and participation in the planning, evaluation, and provision of services [7]. As any other cultural change, this shift is accompanied by enthusiasm, resistance, diversification of opinion, and a need for negotiation [8].

Over the past decades, two contemporaneous trends have led toward increasing client involvement: the first concerns involving clients in their own care in social and health care services, and the second involving clients in planning and developing services. Traditionally, professionals have made decisions on the basis of their medical knowledge, relying on what they deem best for the client without really involving them in the decision-making process [9]. In this “paternalistic care philosophy” the role of the client has mainly been restricted to expressing their agreement with the professional’s decision [10]. The “consumerist movement” sought to increase clients’ opportunities to decide what services and treatments were most suitable for them [11]. The role of a professional became limited to providing the kind of medical information that a client would not have access to without the professional’s specialized expertise [10]. More recently, client-centeredness has become a key guiding paradigm in social and health care services. Its core idea is to elicit and understand clients’ needs, concerns, and expectations in order to reach a shared understanding of the problem and its treatment [12]. Even today, the client-centered care philosophy emphasizes an equal, collaborative partnership between a professional and a client, thus representing a marked shift from the traditional asymmetric doctor–patient relationship that involved a passive patient and a dominant clinician [13].

Along with gaining power to influence their own care, clients are increasingly encouraged to contribute to the planning, evaluation, and development of the services they use [14]. This is part of the larger development of involving citizens in public policymaking, discussed broadly in, for instance, the fields of service management [15,16,17] and public administration [18,19,20]. Typically, client input has been elicited in a fairly restricted manner, inviting their reactions to specific services in a form of structured feedback survey or a client questionnaire [21]. Nowadays, clients are given a more active role in quality improvement when they are invited to co-develop services in collaboration with professionals. The main idea in co-development is the creation of value through interaction between service providers and users [22]. This trend has created a more equal relationship between clients and professionals as it strives for a genuine dialogue between participants [23].

In addition to leading to more accessible and acceptable provision of services [14], client involvement has been seen as a normative good that is valuable in itself [24]. It is argued to improve democracy and social inclusion by placing clients at the heart of service delivery [24]. From the client’s viewpoint, an equal collaborative partnership between clients and professionals, the maintenance of trust, participation in knowledge production, and shared decision-making are crucial building blocks of involvement [9,25,26]. Clients seem to wish greater involvement in service delivery but they also want professionals to recognize this wish as optional and as varying according to the context, time, and individual situation [9,26]. The professionals, in turn, have been noted as valuing client involvement as such but to be reluctant to adopt it as a guiding clinical practice [27]. In their view, client involvement may be ineffective and too time consuming, and they are concerned that attention will be directed away from “actual client-work” [24,27,28]. In addition, some studies have reported that professionals feel intimidated by the new power relations: greater client empowerment may be experienced as threatening professional boundaries and competencies [27,29].

As noted above, client involvement in their own care and development of services has been studied from multiple perspectives, considering both clients’ and professionals’ views [9,24,26,27]. What has been investigated less is the mutually congruent or discrepant views that the professionals and clients may have on the topic. This would be important to study as the prior research has pointed to the direction that the professionals and clients’ expectations on client involvement may vary [30,31,32]. In this paper, our aim is to investigate the implicit expectations that professionals and clients express when they talk about client involvement. Our aims are:

1. To assess the degree to which social and health care professionals and clients share or differ in their expectations of client involvement.
2. To analyze in detail the content of the social and health care professionals’ and clients expectations, paying spcific attention to where the two participant groups differ.

We assume that the results can increase the overall understanding of the role of clients in social and health care services and thus ultimately help us evaluate the potential of client involvement in developing and providing good quality services.

## 2. Materials and Methods

### 2.1. Methodological Approach

In this paper, we use a combination of methods deriving, on the one hand, from interaction-oriented focus group research [33,34,35,36,37,38,39], and on the other hand, from conversation analysis [40,41,42,43]. This means that we operate at the intersection of the substance of conversation and its interactional dynamics, linking our analysis to both the content of the group members’ utterances and the patterns of interaction that they create, see, e.g., [44].

Previous research on focus groups has shown that, in addition to analyzing the content of the group members’ talk, the researcher may also observe how members of the group interact with one another and use these observations as part of the analysis [33,45,46]. Such observations can help the researcher, for example, “to explore the arguments people use against each other, identify the factors which influence individuals to change their minds and document how facts and stories operate in practice—what ideological work they do” [47] (p. 117). As Morgan [45] (p. 718) has pointed out, there is an “inherent connection between the substantive content of ‘what’ a person says and the interactive dynamics of ‘how’ he or she says those things.” The consideration of this connection is elementary when the topic of “client participation” is discussed in a conversation between professionals and the very clients whose participation is at stake at the level of conversation.

In practice, our analysis we examined those segments of interaction where “client participation” was topicalized and discussed. The investigation was guided by the following three questions:

1. What are the views that immediately mobilize an assertion of consensus among the participants [47] (p. 109)?
2. What are the views that are preceded and followed by explanations and accounts, which demonstrate a need to justify one’s views in front of the other participants (see e.g., [48])?
3. Are some views received with explicit expressions of resistance and moral contempt or implicit expressions of opposition through, for example, silence [47] (p. 110); [49] (p. 172); [50]? What are these views substantially about?

On the basis of these considerations, each segment was analyzed with reference to its level of congruency/discrepancy and the contents and implicit expectations that the participants in each case oriented to (for more details, see Section 2.5).

### 2.2. Materials

Our data consist of interaction among social and health care professionals and clients in co-development workshops. These workshops were part of the “Social and health care professionals as experts on client involvement” project of the Finnish Institute of Occupational Health. The project involves municipal social and health care organizations and aims to promote work practices that enhance clients’ involvement in their own care, as well as in planning and developing services. As part of the project, six regionally comprehensive client-involvement workshops were held in five different social and health care organizations to develop their organizational work practices. The aim of the workshops was to create a shared view of client involvement, identify what needs to be improved, invent small experiments to change work practices, and evaluate these experiments. The workshops were based on expansive learning theory [51], the change-management workshop method [52], and service design. In this study, the data came from the first two workshop processes conducted in two large, municipal social and health care organizations. In the first organization, the workshop process targeted client involvement among clients with mental health problems and substance abuse. In this organization, the workshop meetings were audio-taped. In the second organization, the process focused on first-contact services for elderly clients. This process was video-recorded using one camera located in the corner of the room, and was also audio-taped.

The data thus consisted of audio and video recordings of four three-hour workshops (12 h of interaction). The workshop meetings were organized around group discussion assignments on client involvement. These assignments involved, for instance, defining “client involvement,” creating a map of how client involvement has developed in the organization, and assessing stories of smooth and challenging customer journeys. The workshop participants were divided into small groups of four to five, sitting at round tables. The facilitators initiated the discussions on the assignments by giving instructions. The small groups discussed the assignment freely and made notes, after which each group shared the main point of their discussions with the whole group. Thus, the workshop discussions were relatively loosely structured, and the participants were able to choose how and how much to contribute to the discussions.

### 2.3. Research Subjects

The data consist of 35 different participants. Each of the four workshops had approximately 15 participants: eight to 12 professionals, two to four clients, and two to three facilitators. As the workshops were primary organized to develop organizational work practices, the participants were recruited within the organizations without any research-based inclusion or exclusion criteria. The 25 professionals participating in the workshops were chosen by the managers of the organization (in collaboration with the professionals) to represent different occupational groups working with the client group in question. These occupational groups included nurses (*n* = 7), service advisors (*n* = 8), social workers (*n* = 2), physiotherapists (*n* = 2), development specialists (*n* = 3), and department managers (*n* = 3). Most of the professionals (*n* = 24) were females and only one was a male (a nurse). We do not have the information of their ages and levels of experience but in general they represented the whole spectrum, from young to more experienced professionals. The six clients participating in the workshops had either an ongoing treatment at the organization, or had previously been treated there. Three of the clients were male and three were female. We do not have specific information of their ages, diagnoses or other backgrounds apart from information they told in the workshops. The clients were recruited by the professionals and many of them had already participated in the development of services in some way or another. Some of the clients had also acquired training in expertise of experience by a third sector organization and thus gained a more official role in the organizational development activities. The four facilitators were all females, with a background in social, educational, and health sciences, and had a vast experience in facilitating the organizational development processes. They worked at the Finnish Institute of Occupational Health and the National Institute of Health and Welfare.

### 2.4. Ethics

The study was conducted in accordance with the Declaration of Helsinki, and permission to collect the data was obtained from the health care districts and the Finnish Institute of Occupational Health’s Ethics Committee (23 November 2018, project 3517803). Informed, written consent was obtained from all participants before they participated in the study, and they were advised that they could withdraw their consent at any point during the data collection. All names and other details that could enable identification of the participants have been altered in the text and data excerpts.

### 2.5. Analytic Process

Our interactional data from the co-development workshops were analyzed with methods of conversation analysis [40,41,42,43] and interaction-oriented focus group research [33,34,35,36,37,38,39]. We began our analytical process by watching and listening several times to the recordings, making notes on the segments during which the topic of “client involvement” was discussed. Although the workshop assignments revolved around this very topic, there was a lot of discussion on other related topics as well, such as multiprofessional collaboration. This study, however, is based only on the collection of those segments of interaction where client involvement was the participants’ main topic (*n* = 108). In order to warrant a more detailed analysis of these segments, they were transcribed using the conventions of conversation analysis, which necessitates focus not only on talk, but also on the ways in which the participants’ turns are received by the co-participants on a moment-to-moment basis, whether turn transitions are accompanied by overlap or silence, and whether the participants engage in salient nonverbal behavior in terms of gaze direction, gestures and facial expressions, see [41] (pp. 265–269) and the Appendix A. Thereafter, we started to work with the data-segment collection in a data-driven way, probing the categories and patterns identified in a single data segment against every new segment of data. Later, we tested our intersubjective grasp of these patterns with three analysts’ (E.W., S.K., and L-L.U.) independent coding of pieces of data, which led to several further specifications into categories that we had jointly agreed upon and that we could reliably identify in the data. By focusing on the participants’ ways of receiving and responding to each other’s views in the group, we classified each segment as exhibiting either congruence or discrepancy, and then compared the specific contents of each segment, paying specific attention to the implicit expectations that the participants oriented to between the two participant groups. In so doing, we also identified tensions between the participants’ views on client involvement and the clients’ opportunities to influence the co-development workshop discussion in the here and now. The data extracts presented in this paper are drawn from across our entire data set on the basis of their capacity to demonstrate the between-group differences on which our analysis focuses.

## 3. Results

In the following, we present the results of our qualitative analysis in three sections, each of which focuses on one specific topic in our participants’ talk about client involvement, which arose inductively from our analysis of the empirical data. First, we examine how the members of our workshops discussed the ideal of promoting client involvement, demonstrating the high level of consensus that existed among our participants with regard to the topic. Second, we consider an issue that the participants oriented to as more conflicting: is it enough that clients are heard or should they also have actual power to influence the decisions made regarding their services? Finally, we examine the differences in how clients and professionals oriented to the complex interwovenness of cooperation, authority, and responsibility.

Our analysis thus centers around the question of whether the participants express mutually congruent or discrepant viewpoints when discussing these three topics. To give the reader an overall grasp of the prevalence of these patterns across our entire data set, Table 1 summarizes the numbers of instances of congruence and discrepancy with reference to the three topics. The four columns of the table show these numbers both (1) within the groups of professionals only and (2) within the groups consisting of both professionals and clients.

### 3.1. Client Involvement Should be Promoted

In our workshop data, both the clients and the professionals agreed that, on a general level, client involvement should be promoted by giving the clients more opportunities to influence the development of their services. This topic was often discussed as a future ideal, the main obstacle for its current realization being the health care system. The “system” was seen as a common enemy that neither the clients nor the professionals had power to influence. This will be demonstrated in Extract 1, in which one of the professionals (P1) suggests that client involvement necessitates trust between clients and professionals, but that the professionals have not been given time to build trusting relations with clients, as their employer demands them to have a high number of appointments per day. The excerpt is taken from a small group discussion during the first workshop, in which the participants—consisting of both professionals and clients—are given the task to discuss how they understand the concept of client involvement.

Extract 1.

01 P1: mikä tohon asiakkaan osallistamiseen
*what is needed to make the client involved*
02   tarvitaan niin tota jos on ikääntyneist kyse ni se
*I mean erm if they are elderly people then*
03   et saa sen semmosen luottamuksen siihen ni se vaatii
*gaining that trust requires sufficient*
04   sen ajan et jos se niinku työnantaja sit taas sanoo
*time but if the employer then says that*
05   et pitää olla käyntejä niin ja niin paljo ni sit
*you should have so and so many appointments*
06   toisaalta et se et saa sen luottamuksen asiakkaaseen
*and on the other hand that trust is needed to*
07   ja saa hänet osallistuu ni tota se tarvitsee kyl sen
*get her involved then it really requires a*
08   tietyn ajan.=
  *certain amount of time*.=
09 P2: =kyllä.
  =*yes*.
10 C1: ja tähän liittyy myös se et tuota sitte tää
*and this is also connected to the point that the*
11   palveluntuottaja tai kotipalvelussa henkilö ei vaihdu
*service provider or homecare personnel doesn’t change*
12   tai että lääkäri ei vaihdu tai et (.) sais niinku
*or that a doctor doesn’t change or that (.) so that*
13   tniitten määrättyjen tuttujen [ihmisten kanssa asioida.
  *you could deal with the [same familiar people*.
14 P2: [samat työntekijät.
  *[the same employees*.

In the first lines (1–7), a professional (P1) elaborates on her view on client involvement. She suggests that, in order to get the client involved, a certain amount of trust between the client and the professional is needed. P1 also states that building a trusting relationship necessitates time, which she does not necessarily have. Immediately after her turn, another professional (P2) shows agreement with her view (line 9). Then, a client (C1) takes a turn, which she constructs as a straight continuation of P1′s turn (note the turn-initial connector *ja* “and” in line 10). In C1′s view, the question of trust is further associated with the issue of constantly changing service providers. P2 responds in overlap, suggesting candidate words for her co-participant’s turn completion (line 14). This type of anticipatory co-completion has shown to demonstrate understanding [53] and strong agreement between participants [54].

In sum, both the professionals and clients agreed on the line of action that described obstacles and concerns about the realization of client involvement. They perceived these obstacles as being related to organizational factors that they had no power to influence, such as excessive workload (lines 4–8) and the permanency of the staff (lines 10–14). Thus, when considering client involvement as a future ideal whose realization was out of their hands, the views of the professional and client members of the workshop were mutually congruent.

### 3.2. Being Heard or Exerting Influence?

Listening to the client is commonly considered a critical component of all aspects of social and health care services, e.g., [55]. Understanding the client’s situation and lifeworld relies on the professional’s capacity to listen to the client’s experiences and to respond to what they hear [56]. Being heard during consultations is also something clients seem to desire more than anything else [57,58]. In our workshop discussions, both the clients and the professionals considered the professional listening to the client’s questions and concerns a prerequisite of client involvement. This topic was associated with a relatively high level of apparent consensus. Yet, content-wise, the professionals and clients emphasized slightly different ideas, which points to a subtle discrepancy between the views of these two participant groups. Below, we first show an example how this topic was discussed among the professionals only and then an example of the discussion among both professionals and clients.

When conceptualizing client involvement, the professionals stressed the client’s need to be heard and understood. This pattern is exemplified by Extract 2, which starts by one of the professionals (P1) initiating talk about the question “what is client involvement?” asked by the workshop facilitators, and inviting other small-group members to talk about it.

Extract 2.

01 P1: mutta se että miten niinku (0.5) sitte se
*but then how about like (0.5)*
02   asiakasosallisuus niin,
  *client involvement then*,
03 P2: yks on ihan se et miten tulee kuulluks.
*one thing is how you are heard.*
04 P1: niin ja ymmärretyks sen [tarpeen kanssa,
  *yes and understood in terms of that [need*,
05 P3:             [mm.
06 P2:             [nii,
              *[yes*,
07 P1: että ku ensin on se tarve.
*when first there is that need.*
08 P2: mmm,
09 P3: nni on
*that’s right.*

In lines 1–2, one of the professionals (P1) refers to the assignment (*What is client involvement?*) that the small group is supposed to discuss. She leaves the sentence unfinished (note the turn-final particle *nii* “then”), thus encouraging the other group members to present their views. As a response, another professional (P2) states that one aspect of client involvement is that the client is heard. P1 immediately responds with the particle *nii* “yes,” claiming agreement with the position presented by P2 [59]. P1 also extends P2′s turn by adding another element, the client’s need to “be understood,” to the basic idea. These types of extensions that grammatically complete the previous sentence have shown to display strong mutual engagement and shared understanding of the matter at hand [60]. At this point, both P3 (line 5) and P2 (line 6) produce minimal responses, thus demonstrating their agreement with P1′s view. P1 continues by highlighting that it is this client’s need that the professionals should understand (line 7). Again, both P2 and P3 display agreement with the view (lines 8–9). Thus, there seems to be a strong consensus among the professionals that listening to the client and providing them the experience of being heard is what essentially constitutes client involvement.

The clients, however, raised the possibility that being heard is not the same thing as having actual power to influence the decisions about social and health care services. In this way, the clients invoked the question of an equal (or unequal) relationship between the professional and the client. This is what happens in Extract 3, in which the workshop participants are writing their views on post-it notes and choosing pictures that symbolize client involvement.

Extract 3.

01 P1: voisko lapset kuvata sitä asiaa
*could children illustrate a situation in which*
02   et tulee kuulluks ja nähdyks.
*person is heard and seen.*
03 C1: onks se nyt jos tulee kuulluks ni onks se
*is it then if someone is heard is it then*
04   sama asia ku vaikuttaminen
*the same thing as influencing*
05   et saa vaikuttaa jollain tavalla.
*that one can influence somehow.*
06 P2: joo kyl se niinku joo-o,
*yeah I think uh yeah,*
07 P1: mun mielest kyllä mut sä voit käyttää myös sitä sanaa.
*think yes but you can use that word too.*
08 C1: se osallisuus no nii (.) kohdatuksi samanarvoisena.
*that involvement yes (.) to be considered equal.*
09 P1: kaks viiva kolme kuvakorttii (.) meil on koht
*two to three pictures (.) we’ve used*
10   kaikki otettu käyttöön.
*almost all of them.*

At the beginning of the extract, one of the professionals (P1) proposes a picture with a child on it and suggests that a child could illustrate the experience of being heard (lines 1–2). At that point, a client (C1) takes a turn but, instead of confirming P1′s proposal, he goes back to the professionals’ initial perception that highlighted the importance of being heard and questions if being heard is the same thing as being able to influence things (lines 3–5). The client’s challenge to the professional’s view is implicit in that it is presented in the form of a question, but—importantly—the client still raises the possibility that these two aspects of client participation may not always go hand in hand, which calls into question the emphasis on the professional view. In response to the client’s question, P2 produces a hesitant answer, which action-wise serves as a confirmation that “being heard” and “influencing” could essentially be perceived as the same thing. After this, P1 takes an even stronger position, claiming that, in her view, these two aspects of client participation are the same (line 7). She also concludes by stating that the client can also use the word “influence” (which most likely refers to the participants’ task of writing down their views on a post-it note). By designing her turn as a permission-like “commissive” (see the modal verb *voida* “can,” *sä voit käyttää* “you can use”; [61]) P1 positions herself as someone who has the right to direct the client’s actions in a workshop. After P1′s “permission,” the client suggests that involvement could mean that the client is considered equal (line 9). The professionals do not respond to this client’s suggestion but continue with the agenda of the workshop task.

As demonstrated in Extract 3, the clients displayed an orientation to the expectation of what their role should be, not only that they would be heard with respect to their medical conditions and troubles, but that they would be considered equal to the professionals. Indeed, the notion of being heard is inherently asymmetrical in that it applies only to the clients, portraying them in a somewhat passive position in that their involvement is dependent on the professionals’ ability to understand their situation. What was at stake for the clients, then, was the real power to influence decisions about their services.

### 3.3. Cooperation, Authority, and Responsibility

As pointed out at the beginning of this paper, client involvement is often conceptualized with reference to an equal collaborative partnership between clients and professionals [9,25]. By shifting the distribution of power from professionals to clients, the latter are seen to be empowered with greater influence over the decisions that affect them [62]. The basic assumption is that when participating in making decisions about their own treatment, clients will take more responsibility for their situations and cope better [63]. The importance of responsibility as a result of empowerment was also acknowledged by the members of our workshops. However, while the professionals emphasized the responsibilities of the clients, they nonetheless defended their own right to decide on the suitable treatment for the client. The clients, on the other hand, resisted not only the professionals’ sole decision-making authority, but also their handing over the responsibilities to them. Again, we first show an example of the discussion among the professionals only and then among the professionals and clients.

The professionals stressed the importance of the clients’ ability to cooperate in matters concerning their own care. The professional view did not really present this cooperative relationship as one between equals. Rather, the professionals expressed their frustration with situations in which clients do not understand what is best for them. According to the professionals, the clients should—paradoxically—accept their inability to understand what is best for them and give the decision-making power to the professionals. This orientation is visible in Extract 4, which starts by one of the professionals (P1) describing the challenges associated with a client refusing to adhere to a treatment recommendation by the professional.

Extract 4.

01 P1: on aika haasteellisii tilanteita et kun potilaat ei
*it’s quite challenging when the patients refuse*
02   suostu menee tutkimuksiin eikä suostu ottaa lääkkeitä
*to go to examinations or won’t take their medication*
03   eikä suostu tekee mitään ku ne ei ymmärrä sitä omaa
*or do anything because they don’t understand their own*
04   tilannettaan niin asiathan ei kauheesti etene.
*situation so things won’t really progress.*
05 P2: mmm nii.
*mmm yeah.*
06 P1: eikä lääkärikään kauheest siinä voi auttaa jos potilas
*and the doctors can’t help much if the patient*
07   ei oo yhteistyökykyinen tai kukaan terveydenhuollon
*is incapable of cooperating or no professional*
08   ihminen oikein pysty auttamaan jos ei oo ja se tilanne
*can really help if so and that situation*
09   ei parane välttämättä sitte yhtään [myöskään (.)
*won’t necessary get any better [either (.)*
10 P2:             [mmmm.
11 P1: pitäiskö nyt laittaa sit lappu,
*should we make a note then,*

In lines 1–4, P1 produces a three-part list to describe challenging care-work situations. She first mentions clients refusing to go to referred examinations, secondly refusing to take their medication, and thirdly refusing to do anything, this final “extreme case formulation” serving as a way for the professional to legitimize her claim [64]. She also explains that such problems arise when clients do not understand their own situations (lines 3–4). In this way, the professional implies that the clients actually hinder the progress of their own care (line 4). In line 5, another professional (P2) shows agreement with the view, the particle *nii* “yeah” indicating that she is familiar with this type of situation [59]. P1 continues, stating further that the professional cannot help the client if they are “incapable of cooperating” (lines 6–8). What she seems to be suggesting is that the clients’ cooperation should realize in that they give the professionals the power to decide what is best for them. At this point, P2 minimally agrees, and P1 suggests that they write it down on the post-it note (line 11), thus treating her co-participant’s display of agreement as sufficient [65].

Hence, although they emphasized the importance of cooperation, the professionals still portrayed the client’s role as quite passive. In order to receive adequate treatment and for care to progress, the clients were mainly expected to adhere to the professionals’ recommendations. It was thus suggested that the professionals had the ultimate authority to promote what they consider to be the best for the clients.

Interestingly, however, the professionals also highlighted the need for the clients to take responsibility for their own care. This perspective to the issue is demonstrated in Extract 5, in which the participants discuss and write down their conceptualizations of client involvement. At the beginning of the extract, one of the client members of the workshop highlights the need to make a person become involved (line 1). This is, however, met with a lack of substantial agreement. Instead, the professional participants of the group turn the discussion toward the topic of “responsibility” as one aspect of client involvement (line 4).

Extract 5.

01 C1: onks se myös et osallistetaan,
*is it also that a person is made to get involved,*
02 P1: kyllä sitäki paljon käytetään mut ei se,
*that’s also used a lot but it isn’t,*
03   (0.5)
04 P2: tavallaan kuitenki myös vastuu,
*kind of a responsibility, too*
05 P1: nii joo totta.
*yeah that’s right.*
06 P2: riippuu mit- (0.2) oma vastuu omast itestäki.
*it depends wha- (0.2) responsibility for oneself.*
07 P1: joo.
*yes.*
08 P2: mä nyt laitan sen tähän mukaan.
*I’ll put it on here now.*
09 C1: ymmärretyksi tuleminen
*being understood.*

After the somewhat ambivalent reaction to the client’s proposal (line 2), P2 suggests the idea that client involvement also involves responsibility. This idea is immediately supported by P1 (yeah that’s right, line 5). At this point it is not yet entirely clear what the term “responsibility” entails, but in line 6, P2 makes it clear that she is talking about one’s responsibility for oneself. In this case, the implication is that the client takes responsibility for their own situation and care. Compared to the viewpoint of the same professionals expressed in Extract 4, this idea is radically different. Now the clients are seen in the active role of empowered actors, who have control over their lives. After P1′s agreement (line 7), P2 displays an orientation to a sufficient level of consensus among the participants by announcing that she will write it down on the note (line 8), see [65]. At this point, however, one of the clients (C1) takes a turn and expresses a different viewpoint: the client suggests the phrase “being understood” as an alternative conceptualization for client involvement. Thus, quite interestingly, when the professionals’ “unspoken” alternative was to consider clients as influential decision-makers and the professionals handing responsibility over to them, the clients agreed less and suggested something very different from “taking responsibility.”

As shown above (see Extract 4), the professionals referred to their own responsibility and superior authority when deciding on a suitable treatment for their clients, and the clients abiding by this norm was seen as “collaboration.” Extract 6 below demonstrates that clients also orient towards compliance with professionals’ decisions as the one and only option for them to demonstrate their willingness and ability to cooperate. At the beginning of the extract, one of the professionals (P1) states that in health care it is the doctor who makes the decisions.

Extract 6.

01 P1: kyllähän mun mielest on ihan selvä etteihän ihminen
*I think it’s completely clear that a person can’t*
02   määrittele siis terveydenhoidossa (.) ihminen ei
*determine things I mean in health care (.) people can’t*
03   sinänsä voi määritellä miten häntä hoidetaan (.)
*in general determine how they’re treated (.)*
04   lääkärihän sen päättää lääkäri vastaa siitä
*It’s a doctor who decides a doctor is responsible*
05   mitä voi mut potilas voi hyväksyä sen tai ei tai
*but a patient can accept it or not or*
06   ylipäänsä sitä neuvotellen kannattaa tehä,
*or in general it’s advisable to negotiate,*
07 C1: sit taas jos sä et hyväksy ni sit sä et oo
*then again if you don’t accept it then you’re not*
08   hoitomyönteinen (.) tätä oon kuullu tosi paljon kans.
*a compliant patient (.) this is something I’ve heard a lot too.*
09 P1: joo se varmaan on mut tietyllä tavallahan
*yeah it probably is but somehow*
10   se sit vaan on niin että tietyi asioita on sitten
*it just is so that some things just are like that*
11   sellasii (.) et ihminen ei voi tilata hoitoa ku pizzaa.
*(.) people can’t order a treatment like a pizza.*
12 P2: kaikkee ei voi hoitaa kaikel tapaa et jotku asiat pitää
*everything can’t be treated in every way there are some things*
13   hoitaa tietyl tapaa et ne tulee hoidetuks.
*that have to be treated in a certain way so to that they will be taken care of.*
14 P1: nii et semmosia hoitojuttuja mitkä yleisesti tiedetään
*so certain treatment things that are generally known to be*
15   toimiviks et kyllä tässä se semmonen rajanveto tai
*effective that there is this kind of line to be drawn*
16   käynti että ihminen tulee kuulluks ja saa sanoo oman
*that a person is heard and can state their own*
17   sanansa mutta että tulee sit kuitenkin se hoito tietyl
*opinion but that the treatment is determined*
18   taval määritellyks.
*in a certain way.*
19 C1: nii eihän noi yksinkertaisia asioit oo.
*yes these are not simple things.*
20 P1: ja siitä se kai periaatteessa se puhuminen vast alkaa
*and in principle that’s when the talking begins*
21   jos ollaan kauheen eri mieltä et miten se sit hoidetaan.
*when we really disagree on how it’s to be handled.*
((begins to talk about the organization moving to a new building))

In lines 1–4, P1 makes a strong statement that, in health care, a person cannot determine how they are treated. P1 uses the clitic particle -*hän* (kyllä*hän*, ettei*hän*, line 1), which has been argued to indicate common knowledge [66] and expresses certainty in talk (*ihan selvä* “completely clear”). P1, in other words, presents her view as something that is self-evident. By announcing and reminding others about this state of affairs, P1 also positions herself as more authoritative and knowledgeable than the others. In line 4, P1 further states that it is a doctor who “decides.” However, she immediately corrects her own speech and states that it is a doctor who is “responsible” for the treatment. By this type of self-repair, P1 displays her normative orientation towards what is meant to be talked about in the given context [67]. It seems that in the contemporary “client involvement discourse,” professionals’ responsibilities may well be normatively easier to topicalize than their decision-making authority over the client. Indeed, in lines 5–6, P1 seems to seek to further mitigate her prior view on decision-making, emphasizing that it is not only the professional alone, but the client and professional together, who negotiate treatment decisions. The client’s role in this negotiation is, however, presented as narrow: the client can either accept or refuse the professionals’ decisions (line 5).

At this point, however, one of the clients (C1) states—possibly sarcastically—that if a client does not accept the professional’s decision, then they are not seen as *hoitomyönteinen* “compliant” (lines 7–8). With this statement, the client seems to be referring to the traditional view according to which a “good patient” is passive and compliant, e.g., [68]. Thus, if a client wants to be a “good patient”—that is, to cooperate and play the game with its long-established rules [50,69]—they have no other option but to accept the professional’s decision. In this way, the client expresses doubt about their ability to genuinely have a say in the decisions made in social and health care encounters. The client substantiates his claim by also pointing out that this is something he has heard from others and does not represent (only) his own experience.

In lines 9–11, P1 responds to the client. She reformulates her prior statement by using a figurative expression “people can’t order a treatment like a pizza” (line 11), which, in this context, comes across as highly defensive. These types of expressions have been observed in connection with complaints, for example, to enhance their legitimacy and to bring the complaint sequence to a close in the face of a lack of agreement [70]. At this point, another professional (P2) joins the conversations, supporting P1′s view (lines 12–13). He emphasizes the need to have *hoitojuttuja* “treatment things” managed in a certain way, which means favoring the solutions that have proven to be effective. He also highlights a need to draw the line between a client being heard and stating their opinion, on the one hand, and the professional determining the client’s treatment, on the other (lines 14–18). The client concedes by agreeing that these are not simple things to deal with (line 19), which is followed by P1 pointing out that client disagreements will be dealt with as they occur in the social and health care encounters. With this comment, P1 closes the discussion and moves on to a new topic.

Thus, even if the professional in this situation expressed willingness to attend to clients’ concerns in the future once they become relevant during the consultations, in so doing, she ignored the client’s concern in the here and now of the client involvement workshop encounter. In this case, the client’s concern was on a meta level, being about his theoretical opportunity to have a say in decisions about his own care.

## 4. Discussion

In this paper, we have examined possible differences in the ways in which social and health care professionals and clients in co-development workshops perceived client involvement and unraveled the degree to which they share their perceptions. We found that both the clients and the professionals agreed that on a general level client participation should be promoted, but the main obstacle hindering its realization seemed to be the health care system. When considering client involvement as a future ideal, the realization of which was out of their hands, the views of the professional and client members of the workshop were mutually congruent. Both the clients and the professionals also agreed that being heard was a prerequisite of client involvement. Yet, content-wise, the professionals and clients emphasized slightly different ideas, which points to a subtle discrepancy between their views. The professionals stressed the importance of being heard when conceptualizing client involvement, whereas the clients asked for real power to influence the services. On the other hand, when the professionals handed the main responsibility over to the clients, the clients were not eager to agree with their view. Moreover, in contrast to giving the responsibility of the client’s own care to the clients themselves, the professionals referred to their own responsibility when deciding on a suitable treatment for a client. In this way, they expressed their superior authority to make the decisions. These themes of responsibility and authority were also intertwined with the question concerning collaboration. The clients considered compliance with professionals’ decisions as the only option to display their ability to cooperate. In other words, knowing how to play the “client involvement” game requires recognition of the limitations of that very involvement.

These findings reflect the tensions around “expert” knowledge, control, responsibility, and power traditionally reported in social and health care, e.g., [71,72]. As shown in prior research [9,26], the clients in our data wished greater involvement in service delivery (Extract 3) but they also wanted the professionals to recognize this wish to be optional and varying according to the amount of responsibility the client can take (Extract 5). The clients also considered the participation in shared decision-making as crucial part of involvement [9,25,26] but suspected that being categorized as “non-compliant” prevents them from participating genuinely in decision-making [69]. As noted in Anthony and Crawford’s paper [28], the professionals seemed to value client involvement as such but to be reluctant to adopt it as a guiding clinical practice (as shown in Extract 5). The professionals in Extract 1 referred to systemic barriers for not being able to take their responsibility to make clients involved, and in Extract 5 they laid the responsibility to be involved on the client. It can also be argued that the professionals in Extract 6 present the traditional medical view rather than being adherent to client-centered care or the principles of shared decision making [10,12]. It might be that although the professionals value the client involvement as such, they might experience the greater client empowerment as threatening their professional boundaries [27,29].

In analyzing the ways in which social and health care professionals and clients perceive the notion of client involvement, we found various tensions and discrepancies between their views. It is important to note, however, that unlike in certain conversational contexts, such as radio or television talk shows, in which explicit debates and overt controversies are common and even expected [73,74], people typically avoid argument and disagreement [75,76,77]. This was also the case in our data, in which all the discrepancies analyzed were implicit, occurring below the surface level of the interaction. More specifically, although the participants basically expressed agreement with each other’s views, simply building and elaborating on them in and through the turn-by-turn unfolding of interaction, they displayed differences in their orientations towards how knowledgeable they were, or were expected to be [78] and who was to define what should and what should not be done [79]. Such negotiations consist of participants dealing with each other’s turns, not entirely on their own terms, but in ways that slightly deviate from and refrain from appreciating the full interactional import of the earlier talk [40] (p. 260). The motivation for such negotiations, in turn, may be argued to be simply about the need for people to maintain their views about themselves [79] (p. 383)—in this case, either as clients who have control over their lives and who deserve to be heard and to participate in decisions concerning their own treatment, or as social and health care professionals who have the ultimate authority and responsibility to promote what they consider to be best for the clients. The analysis of the precise contents of these negotiations nonetheless allows us to obtain a deeper understanding of the process of social and cultural change in perceiving the role of the client in social and health care.

The study has a number of limitations that have to be taken into account. We strived to increase the trustworthiness of the qualitative analysis by listening to the recordings while reading the transcript, conducting independent coding of pieces of data and discussing selected segments of the recordings with the research team to specify what we could reliably identify in the data. Another obvious limitation is the relatively small number of participants in our data, which constrains the generalizability of our results. In a similar vein, given that all our data came from a very specific context—client involvement workshops in two Finnish municipal social and health care organizations—our results cannot be freely applied to other contexts. Furthermore, the clients in our data were not randomly chosen; they were particularly active in participating in the organizational development activities and obviously did not represent the heterogeneous group of clients as a whole. It can also be argued that they did not represent the most marginalized and disadvantaged groups of clients. On the other hand, they had personal experience of being in that position, having subsequently also gained the ability to voice their concerns and participate in the “officially” driven development workshops [21].

In addition to the limitations described above, it is also worth noting that the number of the clients participating in the workshops was smaller than the number of the professionals. As the workshops were organized to develop organizational work practices, and the participants were thus recruited within the organizations, it was surprisingly challenging for them to get the clients signed into the workshops. It might be that the actual participation of clients in the co-development of services is still quite scarce, regardless of how big a trend client involvement is in the Finnish social and healthcare services. This imbalance was also visible in our analysis, as we did not have small-group discussions with only clients as participants. The imbalance might have also affected the dynamics of the workshop discussion, as the professionals, who traditionally have the authority to dominate the interaction, were outnumbered. There therefore exists the possibility that this imbalance has reinforced the very power imbalance that the study was set out to examine. However, as we hope has become clear from our analysis, the aim of this study was not assessing the degree to which power imbalances exist or not. Instead, through the means of conversational analytic methodology, our goal was to unravel those nuanced practices of interaction by which power imbalances between professionals and clients are realized in interaction. This, in turn, might have a practical value in informing future co-development processes between professionals and clients.

In terms of clinical practice, our paper highlights the importance of being aware of differing expectations the professionals and clients may have on the client involvement. As these expectations are not easy to negotiate in clinical encounters, some aspects of the client involvement, such as participation in decision-making and taking the responsibility over the care, may be treated as one and the same aspect of client involvement. As this might cause even more confusion and misalignments between the participants, we suggest that the different dimensions of client involvement would not be overly simplified and, as the client in Extract 6 states, treated as simple things.

Our analysis of the “client involvement” workshops has mostly highlighted the differences between the clients and professionals’ views on what client participation entails. In addition to the social and cultural change in the client’s role, such differences also point to a lack of extensive contact between the two participant groups. Although professional–client relations may well be taken into the sphere of meta-level reflection in informal encounters among professionals at the workplace, and clients may have analogous conversations with their friends and family members, our everyday life entails very few situations in which such relations could be discussed by clients and professionals together. The “client involvement” workshops from which our data were collected therefore seem to fulfil an important function in advancing the emergence of a shared understanding of what may be expected from the client. Although in this respect, subtle implicit discrepancies easily escape the eye, our analysis suggests that the participants themselves nonetheless orient towards them. The precise experiential consequences of having to constantly negotiate your self-understandings is an empirical question to be addressed in future research, but a preference may well exist for remarks by recipients that validate the first speakers’ claims of rights to knowledge and decision-making [79,80], while remarks that challenge the speakers’ self-concepts may increase their anxiety [81] (p. 474). Fostering a shared understanding of the role of the client may therefore be a worthwhile goal.

## 5. Conclusions

In this paper, we have analyzed conversations between clients and professionals in social and health care on “client involvement.” As all meta-level reflections in terms of “conversations about conversations,” our data—demonstrating client involvement in talk about client involvement—also showed that what is happening at the level of the content of talk may or may not be in line with what is happening at the level of interaction here and now. When a client in a workshop expresses doubt about the ability of the client to genuinely have a say in the decisions made in the social and health care encounters, a professional—as we saw in Extract 6—may circumvent the client’s criticism by pointing out that client disagreements will certainly be dealt will as they occur in the actual social and health care encounters. Intriguingly, however, by highlighting and drawing on the normative ideal according to which such disagreements cannot be ignored by professionals, the professional actually ignored the concern of the client in situ. It is thus a considerable paradox that in cases such as this it is the client involvement rhetoric and discourses themselves that provide the professionals with resources to actually hinder client involvement.

Allowing client involvement to emerge now (and not in the future) is a critical challenge for any social and health care professional. At the same time, sequences of social interaction are essentially held together by the participants carefully attending to what each of them has just said when designing their responses. Systematically, giving such attention to the client—a phenomenon that some authors have referred to as “nexting” [82,83,84]—allows new insights to emerge, but also implies a degree of lack of control over the outcome of the encounter—something that a professional might not feel comfortable about. Concern over the effective routine functioning of the institution might thus generate a barrier for the professional to engage in practices of “letting the other happen to me” [85] (p. 232). However, determining what truly ethical conduct in social and health care interaction looks like may actually necessitate doing just that.

## Figures and Tables

**Table 1:** The number of interaction segments with mutual congruence/discrepancy of viewpoints in the groups of professionals only and in the groups of both clients and professionals.

	Congruence, Professionals Only	Discrepancy, Professionals Only	Congruence, Clients and Professionals	Discrepancy, Clients and Professionals	Total
Client involvement should be promoted	30	3	24	4	61
Being heard or exerting influence?	6	1	8	8	23
Cooperation, authority, and responsibility	12	2	2	8	24
Total	48	6	34	20	108

## Appendix A

Simplified transcription symbols

[ ] Overlapping talk

(.) A pause of less than 0.2 seconds

(.) Pause: silence measured in seconds and tenths of a second

word Accented sound or syllable

((word)) Transcriber’s comments

- Abrupt cut-off of preceding sound

? Final rising intonation

, Final level intonation

. Final falling intonation

= Continuous talk between speakers
